# Jupiter’s brightest auroras are powered by locally generated Alfvén waves

**DOI:** 10.1126/sciadv.aeg6021

**Published:** 2026-09-16

**Authors:** Ali H. Sulaiman, Barry H. Mauk, Robert L. Lysak, Nicholas S. Kruegler, Yash Sarkango, Jamey R. Szalay, Scott J. Bolton, George Clark, Peter A. Damiano, Wondwossen W. Eshetu, Sadie S. Elliott, William S. Kurth, Evan A. Skinner

**Affiliations:** ^1^ School of Physics and Astronomy, Minnesota Institute for Astrophysics, University of Minnesota, Minneapolis, MN, USA.; ^2^ Johns Hopkins University Applied Physics Laboratory, Laurel, MD, USA.; ^3^ Department of Astrophysical Sciences, Princeton University, Princeton, NJ, USA.; ^4^ Southwest Research Institute, San Antonio, TX, USA.; ^5^ Geophysical Institute, University of Alaska Fairbanks, Fairbanks, AK, USA.; ^6^ Department of Physics and Astronomy, University of Iowa, Iowa City, IA, USA.

## Abstract

Jupiter displays the brightest auroras in the solar system, driven by intensely varying energetic electrons that accelerate into the atmosphere. Here, we provide a comprehensive formalism that accurately describes the bimodality of observed auroral electron acceleration regimes. We demonstrate that the occurrence of the two dominant auroral regimes—broadband and monoenergetic—is dictated by specific characteristic plasma length scales, namely, the relationship between the transverse length scale and the electron inertial length. Furthermore, in contrast to the long-standing acceptance that Alfvén waves responsible for auroral electron acceleration exclusively originate at distant equatorial regions, we demonstrate that they can be locally generated at Jupiter’s low altitudes. From this understanding, we find that locally generated, low-altitude Alfvén waves can be directly responsible for accelerating the most intense auroral electrons at Jupiter. These findings establish that the low-altitude acceleration region can itself become a source of Alfvén waves rather than merely a sink for wave energy, thus altering the standard picture of global magnetospheric energy transfer in strongly magnetized planets.

## INTRODUCTION

Before the arrival of NASA’s Juno spacecraft, the first polar orbiter around Jupiter, the observed brightness and location of Jupiter’s main auroral oval were explained by a steady-state magnetosphere-ionosphere coupling model. Here, the breakdown of plasma corotation in the middle magnetosphere gives rise to electric currents that limit the subcorotation via the transfer of angular momentum from the polar ionosphere. Electrons flowing as charge carriers precipitate into the polar ionosphere leading to auroral emissions. Originally developed for Earth’s aurora (*1*), a relationship between field-aligned current and potential drop was extrapolated for Jupiter [e.g., (*2*, *3*)]. It was naturally hypothesized that the electron distributions above Jupiter that power the aurora would be peaked in energy (monoenergetic). This would be a consequence of electrons unidirectionally gaining a discrete amount of energy, *q*φ_||_, as they fall through the potential drop and are accelerated downward into Jupiter’s ionosphere.

However, early Juno observations above the auroral zones revealed the prevalence of electrons exhibiting broadband-in-energy distributions along low-altitude auroral field lines (*4*, *5*). Subsequent statistics showed that the vast majority of field-aligned electron distributions are broadband—as opposed to peaked—in energy (*6*). This brought to the fore the role of wave-particle interactions in explaining the broadband distributions. Two candidates were proposed: (i) inertial Alfvén waves that interact with electrons at scales where the parallel electric field becomes large (*7*–*12*), and (ii) whistler-mode waves that accelerate electrons via a stochastic process (*13*–*16*). The recent revelation that the auroral ionosphere is significantly and persistently depleted tips the balance in favor of Alfvénic acceleration (*17*). The whistler mode cannot propagate above the electron plasma frequency and would therefore be suppressed in a low-density medium. On the other hand, the capacity of an inertial Alfvén wave to develop a parallel electric field is enhanced at low electron densities. When kinetic effects are considered, electron inertia becomes important. The resulting finite response time of electrons means they can no longer instantaneously short out electric fields parallel to the magnetic field; thus, ideal magnetohydrodynamics no longer holds. In the inertial limit, the Alfvén wave dispersion relation becomes

$${\left(\frac{\omega }{k_{\|}c_{A}}\right)}^{2}=\frac{1+k_{⊥}^{2}\rho_{i}^{2}}{1+k_{⊥}^{2}\lambda_{e}^{2}}\approx \frac{1}{1+k_{⊥}^{2}\lambda_{e}^{2}}$$ (1)

where ω is the angular frequency, $k_{\|,⟂}$ is the parallel and perpendicular angular wave number, respectively, *c*_A_ = *V*_A_/(1 + *V*_A_^2^/*c*^2^)^1/2^ is the Alfvén speed, *V*_A_, modified by the displacement current, λ_e_ is the electron inertial length, and ρ_i_ is the ion gyroradius. When β < *m*_e_/*m*_p_, where β is the ratio of the thermal to magnetic pressures and *m*_e_/*m*_p_ is the electron-to-proton mass ratio, *k*_⟂_^2^ρ_i_^2^ in Eq. 1 can be ignored. With β ∼ 10^−8^, this condition is well satisfied in Jupiter’s low-altitude auroral zones.

The main auroral oval at Jupiter is supported by both upward and downward current regions, named Zone I and Zone II, respectively (*18*, *19*). An unexpected feature at Jupiter is the presence of bright auroral emissions in the downward current region (Zone II), which is found to carry significant downward electron fluxes despite the current polarity. The presence and importance of Alfvénic acceleration at Jupiter remain largely assumed, albeit with robust theoretical argument. Transverse fluctuations in the low-frequency (0.2 to 5 Hz) magnetic field that have been interpreted as Alfvénic were found to demonstrably map to the diffuse aurora, equatorward of Zones I and II. Similar signatures were also reported at very high altitudes >10 *R*_J_ (*20*) where there is ambiguity in magnetic field line mapping to the main auroral zones. Attempts to explain the absence of these fluctuations above the main auroral zones include efficient dissipation (*19*), limitations in instrument sensitivity (*21*), and the increasing electrostatic nature of the inertial Alfvén wave (thus low signal in the magnetic fluctuations). Furthermore, Alfvén waves in Jupiter’s high latitudes are thought to originate exclusively from the equatorial plasma sheet likely due to turbulence mediated by radial plasma flows (*7*) and/or instabilities triggered by the large temperature anisotropies (*22*). This is distinct from the Alfvén waves along the Io-Jupiter flux tube that have a known source, originating as magnetic deformations of wavelength comparable to Io’s extended diameter (*23*–*25*). Because the maximum theoretical frequency of the Alfvén wave is the proton cyclotron frequency, which is ∼1 Hz at 30 *R*_J_ in the equator, it is not possible to definitively relate Alfvén-like fluctuations at the high latitudes to an equatorial origin using measurements from Juno’s plasma wave instrument because 50 Hz is the lowest frequency at which both **E** and **B** can be measured simultaneously. By contrast, locally generated low-altitude Alfvén waves will propagate up to a maximum frequency of order kilohertz, the proton cyclotron frequency in the polar region. Although this is well within the frequency range of Juno’s plasma wave instrument, the absence of multipoint measurements limits examining the dependence of **E** and **B** over *k*-space. That said, dispersive proton cyclotron waves are frequently measured above Jupiter’s auroral zones, which are likely the high-frequency extension of locally generated Alfvén waves (*19*, *26*, *27*), though this evidence should be treated as circumstantial rather than direct.

## RESULTS

**Fig. 1.**
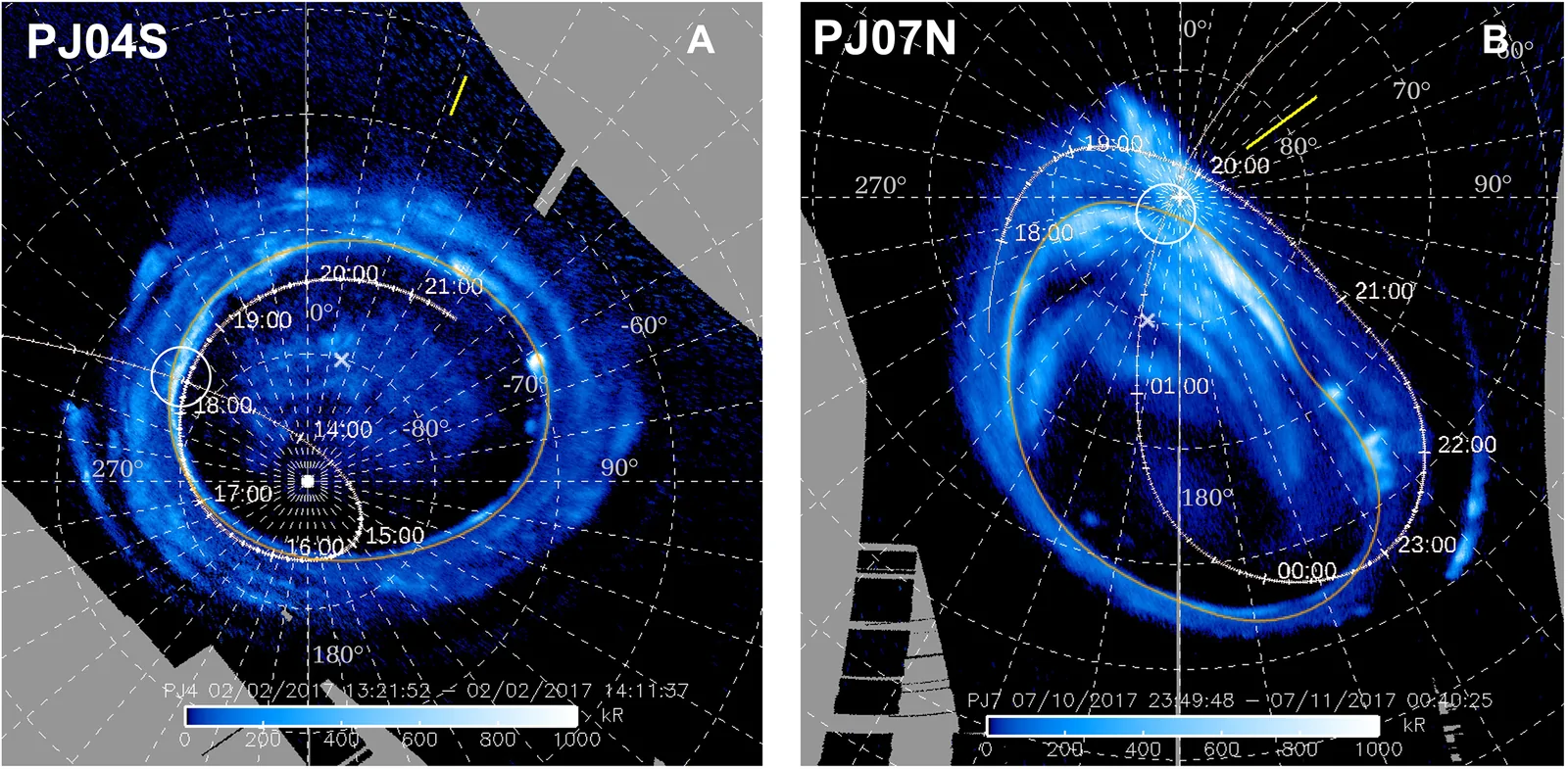
Ultraviolet images of Jupiter’s aurora. Orthographic projections of ultraviolet brightness image ensembles captured by the Ultraviolet Spectrograph (UVS) (*28*, *56*, *57*) for (**A**) PJ4S and (**B**) PJ7N. Overlaid are Juno’s magnetically mapped trajectory in white and the statistical main auroral oval in orange (*58*). The magnetic pole is labeled with a cross. The main auroral crossings analyzed are highlighted by white rings. The yellow lines point along the solar direction. There are nonconstant time delays between when in situ data measurements are recorded and when UV images are captured. This figure is therefore presented for context.

**Fig. 2.**
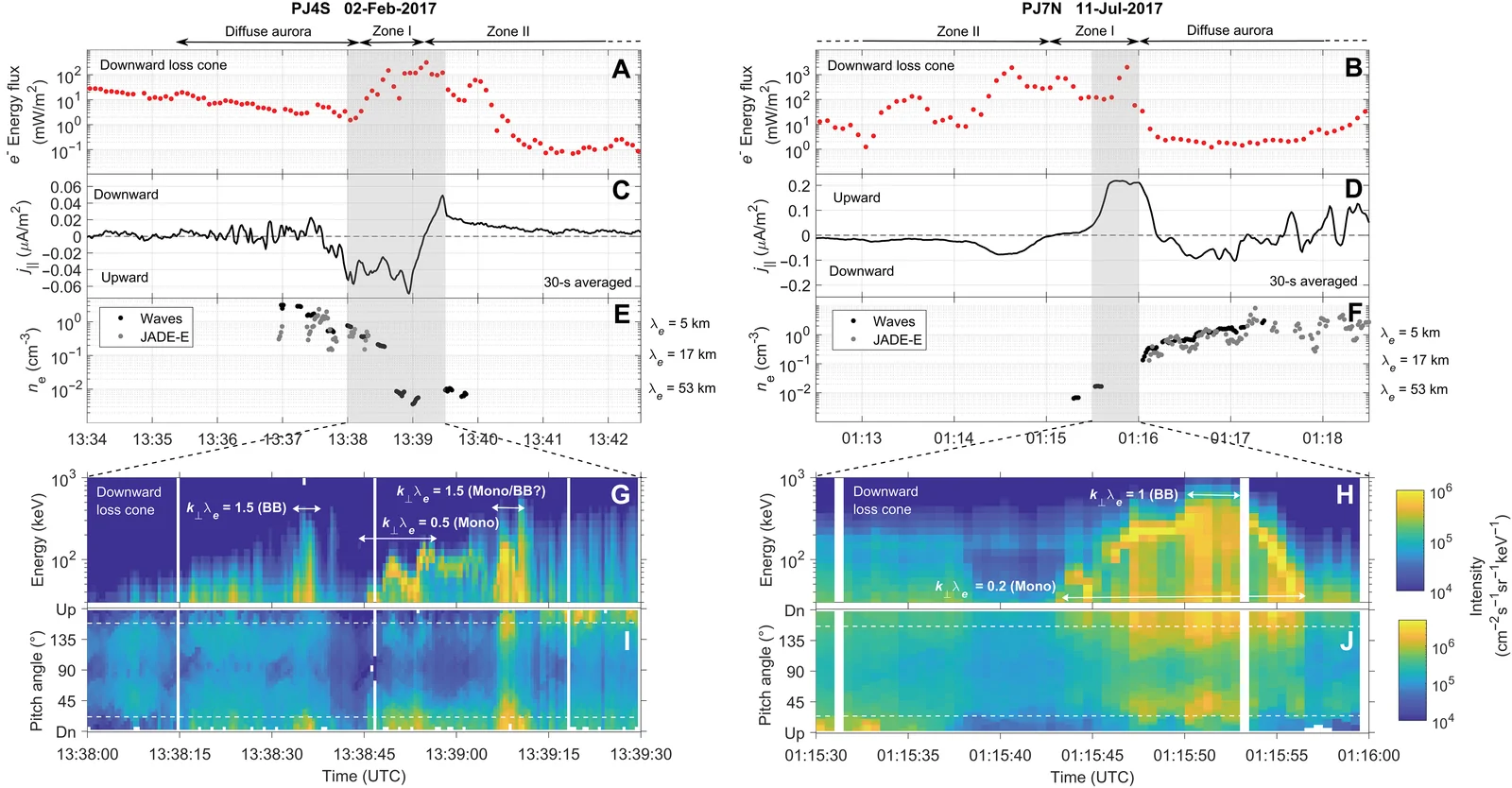
Multi-instrument datasets during Juno’s fourth (southern) and seventh (northern) low-altitude auroral passes. (**A** and **B**) Electron energy fluxes in the downward loss cone measured by the Jupiter Energetic Particle Detector Instrument (JEDI) over the energy range 30 to 1000 keV. (**C** and **D**) Field-aligned current densities calculated from 30-s averaged perturbations in the magnetic field measured by the magnetometer. (**E** and **F**) Electron number densities inferred from plasma wave cutoffs measured by the Waves instrument (black) and from moments of the partial electron distribution measured by the JADE (gray). (**G** and **H**) Energy-time spectrograms of energetic electrons in the loss cone measured by JEDI. (**I** and **J**) Pitch-angle time spectrograms of energetic electrons with loss cone angles overlaid (dashed while lines). BB, broadband; Mono, monoenergetic.

Figure 1 shows images of ultraviolet brightness of two events we examine in this case study. These are assembled from images captured by Juno’s Ultraviolet Spectrograph instrument (*28*) during the fourth southern and seventh northern auroral passes (PJ4S and PJ7N, respectively). Overlaid are Juno’s magnetic footprint track spaced by 1 min. Figure 2 shows the associated in-situ multi-instrument data recorded during these passes. Figure 2 (A and B) show 30- to 1000-keV electron energy fluxes in the downward loss cone. Figure 2 (C and D) show the field-aligned current densities, *j*_||_, derived from 30-s averaged background-subtracted magnetic field perturbations measured by the magnetometer (*29*). This dataset is reliable in determining when Juno crosses the main aurora, particularly in separating the upward (Zone I) and downward (Zone II) current regions (see Materials and Methods). Figure 2 (E and F) show the electron densities, *n*_e_, and corresponding electron inertial lengths, λ_e_, inferred from the particle distributions and plasma wave cutoffs. Equatorward of the main aurora, the majority of data points are from the Jovian Auroral Distributions Experiment (JADE) (*30*, *31*), whereas more poleward data points, including within the main aurora, are obtained from the low-frequency cutoff of the ordinary-mode emission measured by the Waves instrument (*17*, *32*–*34*). Where auroral electron acceleration takes place, the plasma is strongly magnetized, with *f*_pe_ ≪ *f*_ce_, and significantly depleted with *n*_e_ of order 10^−2^ cm^−3^ and λ_e_ of order 10 km. Figure 2 (G and H, and I and J, respectively) shows energy and pitch angle distributions of 30 to 1000 keV measured by the Jupiter Energetic-particle Detector Instrument (JEDI) (*35*). The loss cone angles are calculated and overlaid on the pitch angle distributions. We restrict the JEDI data to subintervals where Juno crossed the main auroral zones.

The PJ4S event shows energetic electron distributions that are typical for Jupiter’s auroral zones. This event is typical of what is observed, whereby the currents are supported by both monoenergetic and broadband acceleration processes, the latter representing the majority of the observed electron distributions (*6*). The monoenergetic distributions are understood to be a consequence of electrostatic acceleration by double layer or localized parallel electric fields from field line resonances because they are often associated with unidirectional and field-aligned electrons and protons simultaneously accelerated in opposite directions. The corresponding “inverted-V” features in energy-time are interpreted as spatial variations across a quasi-static potential structure with the apex representing the point where electrons fall through the maximum potential drop. This is notably similar to what is observed above Earth’s auroral oval, albeit with much higher voltages at Jupiter (*36*, *37*). These structures are generally believed to be a part of a large-scale electric current circuit driven by a generator and dissipated by a load (*38*, *39*). At Jupiter, electric currents are demanded to balance the ∇ × **B** imposed by the magnetospheric plasma flows.

The broadband distributions, on the other hand, are associated with bidirectional (sometimes unidirectional) field-aligned electrons with no evidence of simultaneous field-aligned ion acceleration. These two distributions have been extensively studied at Jupiter, and it has been shown that both appear to have a similar energy flux versus characteristic energy relationship, resulting in either being able to broadly account for the observed UV brightness and color ratio (*40*). This underlines the paraphrased question posed by Mauk *et al.* (*41*): Why is either process favored at any one time? As shown in Fig. 2 for PJ4S, although these two distinct distributions can occur within the same current region (same polarity) and current density magnitude, the local plasma conditions exhibit very strong spatial variability. Between the first broadband distribution at 13:38:35 and the inverted-V at 13:38:49, the electron density falls steeply from 0.20 to 0.01 cm^−3^ with a relatively steady current. While the density remains steady between the inverted-V and the second (predominantly) broadband distribution at 13:39:08, the current reverses polarity. This complicates an examination seeking to explain the preference of one distribution over the other.

PJ7N is a rare event where these two acceleration processes appear to act in concert and their distributions are well separated, as shown in Fig. 2H, which has no precedent within Earth’s auroral regions (*18*, *42*, *43*). This makes the event naturally valuable to examine the underlying onset mechanism for Jupiter’s broadband distributions. Figure 2J shows that the electrons are predominantly accelerated in the downward loss cone with a maximum downward energy flux during the broadband interval. The upward loss cone is largely empty, although there is a population just outside the loss cone mostly during the broadband interval. The entire event coexists within an upward field-aligned current with a quasi-steady magnitude. The density measurements are intermittent in this region with a gap ranging from 0.02 to 0.12 cm^−3^ over 25 s. Interpolating log-linearly over the interval of the inverted-V, which occupies the broadest region where the acceleration occurs, we estimate a density ranging between 0.05 and 0.10 cm^−3^ with a value of 0.07 cm^−3^ in the broadband subinterval. The magnetic field is measured at 170 μT.

One question addressed by Mauk *et al.* (*41*) was on whether the broadband distribution occurred simultaneously with the inverted-V feature, i.e., the double layer was preserved throughout, or whether there was a transition from one mode to another. Inspection of the ion pitch angle distribution during this event revealed that ions were electrostatically accelerated upward where the inverted-V was observed, lending credence to the existence of a double layer with converging electric field. However, where the broadband acceleration was observed, the upward ions appeared to be suppressed and exhibited a conic distribution in pitch angle. This led to the conclusion that the broadband acceleration mechanism was replacing, rather than coexisting with, monoenergetic acceleration (see fig. S1). Furthermore, the broadband acceleration delivered the most intense electron energy fluxes of ∼3000 mW/m^2^ (Fig. 2B). The existence of the broadband distribution within what would have been the apex (or maximum energy) of the inverted-V suggests an instability triggered by an energy threshold of 300 keV being exceeded.

### Inertial Alfvén wave theory

**Fig. 3.**
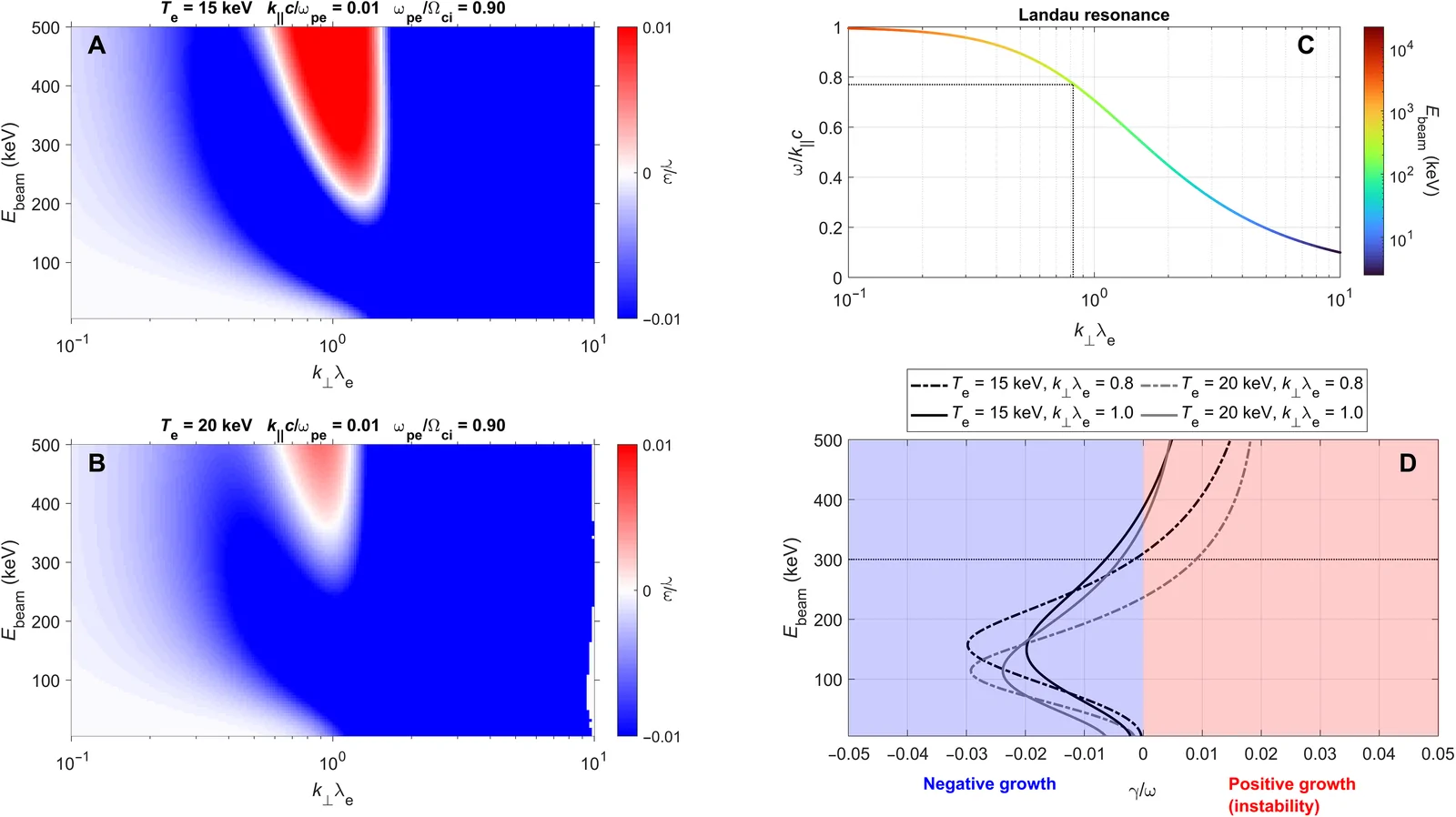
Normalized growth rates for PJ7N event. Calculated for beam energy versus $k_{⟂}$λ_e_ for (**A**) *T*_e_ = 15 keV and (**B**) 20 keV. (**C**) Phase speed versus *k*_⟂_λ_e_ from the inertial Alfvén wave dispersion relation in Eq. 1. The phase speed that matches a beam speed of 0.77*c* or 300 keV is indicated. (**D**) Beam energy versus normalized growth rates with 300-keV beam energy overlaid. The red region is that of instability (positive growth rate).

We begin by making some calculations using cold plasma theory and invoking Landau resonance where ω/*k*_||_ = *v*_beam_. Using Eq. 1 and as shown in Fig. 3C, we replace the left-hand side with *v*_beam_ = 0.77*c* (which corresponds to an energy of 300 keV with relativistic correction) and *c*_A_ = 0.9998*c* to obtain $k_{⟂}$λ_e_ = 0.82. A density of 0.07 cm^−3^ corresponds to λ_e_ = 20 km, which in turn yields $k_{⟂}$ = 0.0415 km^−1^ or a perpendicular length scale, 2π/$k_{⟂}$ = 151 km.

Next, we compare this to the distance traversed by Juno across the broadband region—that is, an estimate of the perpendicular length scale—by multiplying Juno’s perpendicular velocity with the transit time. This gives a comparable value 0.05 km^−1^ or 120 km. This corresponds to $k_{⟂}$λ_e_ = 1. By contrast, the inverted-V feature corresponds to $k_{⟂}$λ_e_ = 0.2.

We now consider the polarization relation of an inertial Alfvén wave, whereby the parallel electric field and the electric-to-magnetic field ratio are given by

$$E_{\|}=\frac{k_{\|}k_{⊥}\lambda_{e}^{2}}{1+k_{⊥}^{2}\lambda_{e}^{2}}E_{⊥}$$ (2)

$$\frac{E_{⊥}}{B_{⊥}}=c_{A}{\left(1+k_{⊥}^{2}\lambda_{e}^{2}\right)}^{1/2}$$ (3)

Because λ_e_ = *c*/ω_pe_, where *c* is the speed of light and ω_pe_ = (*n*_e_*e*^2^/ε_0_*m*_e_)^1/2^ is the electron plasma frequency, it is easy to see that a low electron density, *n*_e_, is conducive to the development of a parallel electric field and hence field-aligned electron acceleration.

We estimate the voltage delivered by an inertial Alfvén wave by combining Eqs. 2 and 3 to get

$${\Delta \varphi }_{\|}=B_{⊥}c_{A}\lambda_{e}\frac{k_{⊥}\lambda_{e}}{{\left(1+k_{⊥}^{2}\lambda_{e}^{2}\right)}^{1/2}}$$ (4)

Using the known quantities and the measured $B_{⟂}$ = ∼100 nT, we obtain a maximum potential drop of Δφ = 380 kV, which is fully consistent with the observed high broadband energies. Together, these results strongly suggest that the inertial Alfvén wave is locally generated by monoenergetic accelerated electron beams that exceed 300 keV.

The waves propagate close to the speed of light, *c*_A_ ∼ *c*, and can therefore only nonresonantly interact with slower electrons (*44*). Consequently, in an inhomogeneous plasma (such as the auroral flux tube), each electron in its rest frame will respond to an instantaneous parallel electric field and will be accelerated or decelerated along the magnetic field during the partial wave cycle before the wave escapes. As a result, this would manifest as a broadband-in-energy and bidirectional distribution, as is often observed in Jupiter’s auroral zones.

### Beam-plasma instability

To further test this argument, we consider the effects of finite temperature, i.e., beyond cold plasma theory, to determine whether the observed $k_{⟂}$λ_e_ and beam speed can lead to positive growth rates (instability). Figure 3 (A and B) displays the normalized growth rates from a kinetic dispersion solver (see Materials and Methods). Specifically, we reproduce most of the plasma conditions of Fig. 2B with the interpolated density and measured magnetic field strength giving ω_pe_/Ω_ci_ = 0.9, where Ω_ci_ is the ion (H^+^) cyclotron frequency.

The electron temperature, *T*_e_, cannot be obtained above the main aurora due to low signal-to-noise ratio, though it was measured up to its equatorward edge and was found to increase poleward, reaching 13 keV at 1:15:55 (*31*). Accordingly, this value is treated as a lower limit because Juno was further poleward before that time. We solve for *T*_e_ = 15 keV (Fig. 3A) and 20 keV (Fig. 3B). An electron beam, modeled as a drifting Maxwellian, is injected into the background population.

The results show that the regions of instability are bounded by $k_{⟂}$λ_e_ ∼ 1 and a minimum beam energy that is dependent on and increases with *T*_e_. Figure 3D examines this more closely by plotting beam energies ranging from 50 to 500 keV against normalized growth rates. For comparison, the observed beam energy threshold of 300 keV from Fig. 2H is overlaid. We examine the growth rates for four cases with *T*_e_ = 15 and 20 keV and $k_{⟂}$λ_e_ = 0.8 and 1. Overall, we find the best case, i.e., where the growth rates become positive when the minimum energy threshold is met to be for *T*_e_ = 15 keV and $k_{⟂}$λ_e_ = 0.8. The three other cases are also reasonably close and are broadly consistent with our estimates of *T*_e_ and $k_{⟂}$λ_e_ measured by Juno.

A similar event occurred during the PJ10N auroral pass (*41*); however, poorly resolved densities and field-aligned current preclude a similarly careful analysis. One of the PJ4S signatures, occurring at 13:39:08, is ambiguous in that the broadband distribution might be perceived as appearing simultaneously at the apex of a narrow inverted-V. The presence of the inverted-V cannot be definitively verified due to the relatively low resolution, and the ion data does not provide additional clues. That said, if this were indeed an inverted-V, we can demonstrate that it would be unstable at the apex, which is >100 keV in this event. During PJ4S, *T*_e_ was found to be roughly half of PJ7N’s, similarly increasing poleward and reaching 7.5 keV at the equatorward edge of Zone I. It is clear from Fig. 3 (A and B) that the beam energy threshold decreases with lower *T*_e_. Calculating growth rates (fig. S2) for *T*_e_ = 10 keV and $k_{⟂}$λ_e_ = 1.5, we find the beam energy threshold for positive growth to be ∼100 keV. This would also explain the pristine inverted-V at 13:38:49, which has a peak energy at 100 keV. This potential structure occurs over a much broader length scale of $k_{⟂}$λ_e_ = 0.5 and an associated beam energy threshold of ∼500 keV. Thus, the potential structure remains stable.

## DISCUSSION

Our analysis combining multi-instrument data, theory, and modeling, enabled by accurate density measurements, confirms that the inertial Alfvén wave can be responsible for the observed broadband distributions that deliver the most intense auroras at Jupiter. Previous work that has interpreted transverse magnetic field fluctuations as Alfvénic (*45*) were found to map equatorward of the main aurora (*19*, *21*). While the role of Alfvén waves has been recognized as important to explain auroral energization, they were hypothesized to have their origin in the equatorial middle magnetosphere and propagate to low auroral altitudes. This may be plausible but is yet to be observationally corroborated. We show that a beam instability in the low altitudes can lead to the Alfvén waves being locally generated. We demonstrate that the interval over which the broadband distributions occur is over a length scale where $k_{⟂}$λ_e_ ≳ 1, the relevant scale for the inertial Alfvén wave to develop a parallel electric field, whereas the monoenergetic/inverted-V distributions are associated with $k_{⟂}$λ_e_ ≪ 1. Because of the number of factors that affect the underlying physics, namely, magnetic field, current magnitude, electron density, electron temperature, and beam energy, we focused our analysis on the PJ7N event where both monoenergetic and broadband distributions coexisted and have the highest quality measurements. This “bimodal” event was brought to the fore by Mauk *et al.* (*41*), though they speculated that the onset of the instability may have been due to the current becoming too high. Figure 2 (C and D) shows that this is not necessarily the case.

**Fig. 4.**
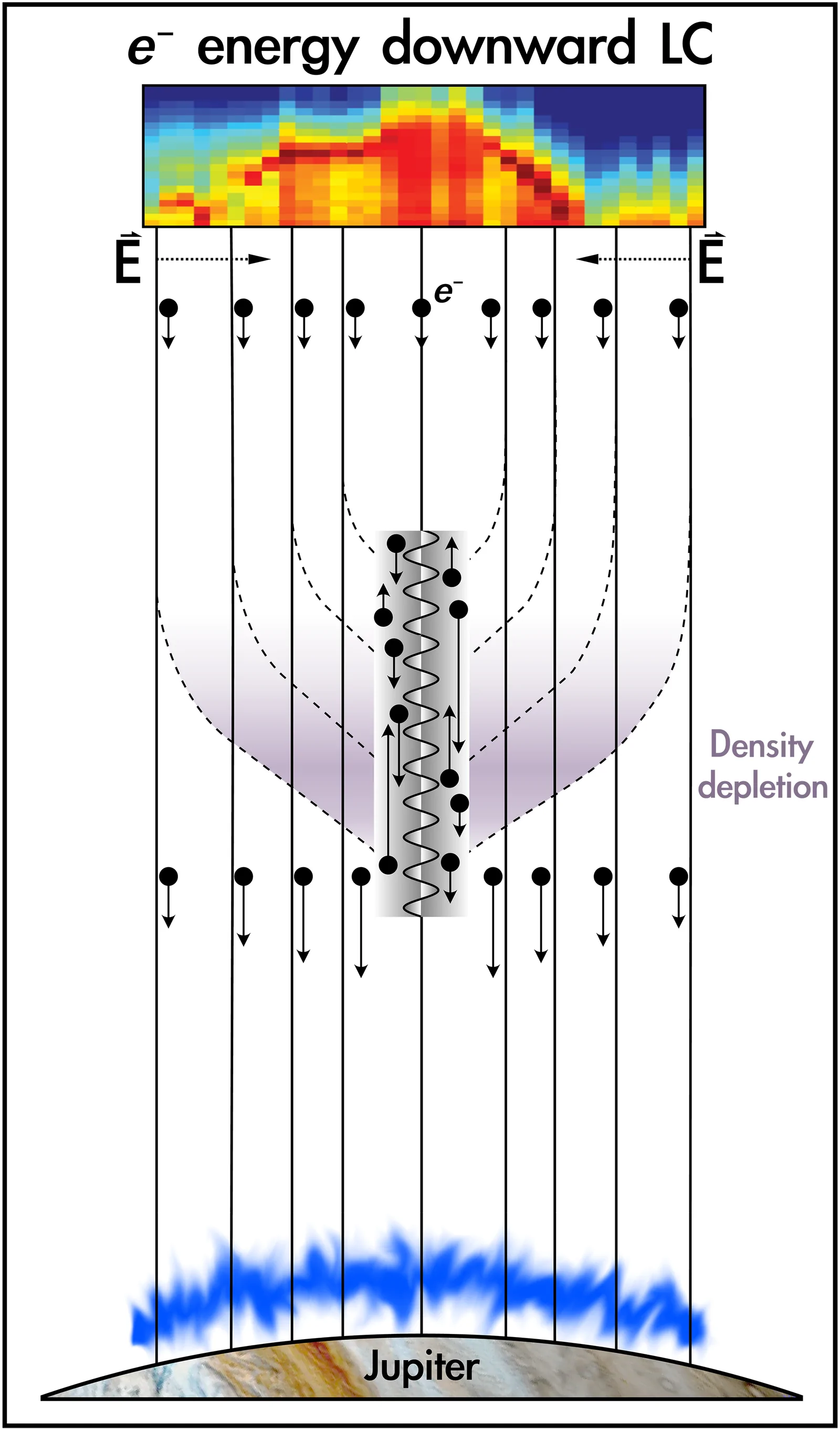
Illustration of the PJ7N auroral acceleration event. This relates the observed electron energy distribution to a spatial structure. The inverted-V signature is associated with a quasi-static potential structure (dashed contours) that has a converging electric field, i.e., an upward field-aligned electric field that accelerates electron downward. The magnitude of the potential drop increases toward the center/apex. Where the accelerated electron energy increases above a threshold, an instability is triggered, which gives rise to an inertial Alfvén wave via Landau resonance. The Alfvén wave propagates close to the speed of light along the magnetic field. A nonresonant interaction with the electrons leads to a broadband and bidirectional distribution. Jupiter image credit: NASA/JPL-Caltech/SwRI/MSSS.

Last, we address the question posed by Mauk *et al.* (*41*) of why the system chooses one mechanism (monoenergetic versus broadband acceleration) over another at any one time or position. We conclude that they are not independent mechanisms, but that monoenergetic acceleration can be a prerequisite for the broadband acceleration. Figure 4 is a graphical illustration summarizing our interpretation. The formation of a double layer is well understood in a collisionless, magnetized plasma where the available charge carriers (electrons) cannot smoothly supply the required current (*1*). The demand for a current in Jupiter’s magnetosphere is imposed by subcorotating equatorial flows (*46*) and/or discontinuous flux tube-driven transport giving rise to local stress imbalance of the magnetosphere-ionosphere system (*7*). The monoenergetic potential drop can be sustained by a double layer and/or global field line resonances creating a localized parallel electric field (*47*, *48*).

Whether the monoenergetic accelerated field-aligned electron beams set up an instability depends on a number of factors in the low-altitude auroral zones, primarily electron temperature, which sets the minimum beam energy threshold, and density, which sets the electron inertial length. Once both conditions are met, locally generated inertial Alfvén waves nonresonantly accelerate electrons to the highest energy fluxes. An inverted-V distribution in isolation would mean the instability conditions have not been met. Such distributions, when fully formed (such as in Fig. 2G), have lower maximum energies of ∼100 keV, indicating that a beam energy threshold is likely not attained and are observed over a longer spatial scale or $k_{⟂}$λ_e_ ≪ 1. These represent regions of negative growth in the beam energy versus $k_{⟂}$λ_e_ parameter space. Conversely, a broadband distribution in isolation has two possible sources. The first possibility could be that the conditions have been met and indeed they are often observed over shorter spatial scales or $k_{⟂}$λ_e_ ≳ 1 with the preexisting beam presumably originating from a double layer structure at altitudes not local to the spacecraft. It is important to recognize that although double layers can move along the auroral magnetic field over a range of altitudes, their parallel electric fields are highly localized. Thus, they are only observed when Juno crosses the auroral zones at an altitude where the double layer is located at the time. The second possibility could be acceleration by dispersive Alfvén waves originating from the equatorial plasma sheet, though this remains to be observationally validated at Jupiter.

In this study, we developed a framework describing the bimodality of auroral electron energy distributions observed at Jupiter and showed that their occurrence is fundamentally controlled by characteristic plasma length scales, specifically the relationship between the transverse scale size and the electron inertial length. Our results further demonstrate that inertial Alfvén waves can be generated locally within the low-altitude auroral acceleration region itself, in contrast to the conventional picture in which auroral Alfvén waves originate exclusively in distant equatorial regions. We find that these locally generated waves can directly accelerate electrons to the highest observed auroral energy fluxes, thereby linking kinetic-scale plasma processes to macroscopic auroral brightness. Together, these findings indicate that the low-altitude auroral region acts not only as a sink of incoming wave energy but also as an active source of Alfvénic activity, fundamentally revising the standard view of magnetospheric energy transfer at Jupiter and potentially in other strongly magnetized plasma environments.

## MATERIALS AND METHODS

### Energy flux

This is computed for electrons in the energy range 30 to 1000 keV within the downward loss cone as

$$\text{Energy flux}=\pi \int_{E_{\text{min}}}^{E_{\text{max}}}I\cdot E\mathrm{dE}$$ (5)

where *I* is the particle intensity (cm^−2^ s^−1^ sr^−1^ keV^−1^), *E* is the electron energy (keV), and π is the area-projected-weighted size of the loss cone. The width of the loss cone is estimated, assuming a dipole magnetic field configuration, as arcsin(1/*R*^3^)^1/2^, where *R* is the Jovicentric distance in Jovian radii.

### Field-aligned current

We estimate the magnitude and polarity of field-aligned currents from measured magnetic field perturbations using Ampère’s law based on the method from (*49*). The magnetic field perturbation is calculated as $\delta B=B_{\text{MAG}}-B_{\text{JRM}33}$ (*50*) using the 1 vector/s cadence MAG measurement $B_{\text{MAG}}$ and the model magnetic field $B_{\text{JRM}33}$. The perturbation field is averaged over Juno’s 30-s spin period to remove remnant spin modulation effects. The model magnetic field is determined from the time-independent internal and external magnetic field models, JRM33 up to order and degree 13 and Con2020 (*51*, *52*), as implemented by the JupiterMag Python wrapper (*53*).

Ampère’s law is applied within a right-handed, spacecraft-centered coordinate system with $\hat{z}$, $\hat{y}$, and $\hat{x}$ defined by the directions along the local magnetic field $\hat{b}$, the spacecraft velocity perpendicular to the magnetic field $v_{⊥}$, and $v\times \hat{b}$, respectively. The one-dimensional nature of the spacecraft trajectory limits the calculation of the curl within Ampère’s law and fundamentally only one of the two terms within the *z* component of the curl can be measured. The inclusion of two terms (by splitting the trajectory into two velocity components), such as has been done previously (*17*), introduces the risk that the current magnitude is artificially and nonuniformly enhanced. Following this discussion, the field-aligned current density $j_{\|}$ is given by

$$j_{\|}=-\frac{1}{\mu_{0}}\frac{\Delta \delta B_{v\times \hat{b}}}{\Delta x_{v_{⊥}}}=-\frac{1}{\mu_{0}v_{⊥}}\frac{\Delta \delta B_{v\times \hat{b}}}{\Delta t}$$ (6)

where $\mu_{0}$ is the permeability of free space, $\Delta \delta B_{v\times \hat{b}}$ is the magnetic field perturbation in the $v\times \hat{b}$ direction, $v_{⊥}$ is the magnitude of the spacecraft velocity perpendicular to $\hat{b}$, $x_{v_{⊥}}$ is the spacecraft position in the $v_{⊥}$ direction, and *t* is time. A limitation of single-spacecraft measurements is the inability to separate temporal and spatial dependencies, so magnetic perturbations cannot be uniquely attributed to electric currents. A fundamental assumption in this calculation is therefore that the magnetic field fluctuations are primarily spatial, which is reasonable because these perturbations are observed repeatedly over the auroral zones, thus making the dependence predominantly in latitude. Following this, we estimate the spatial derivative via the measured temporal derivative in the second form of the equation. It is also important to note that the current magnitudes calculated from this method may be underestimated because we assume the spacecraft is passing orthogonally through an infinite current sheet. In cases with an oblique crossing, the magnetic field perturbation created by the current sheet is not fully probed by the $v\times \hat{b}$ component of the magnetic field.

### Kinetic dispersion relation

We use the formalism laid out by Lysak and Lotko (*54*, *55*). Below the ion (proton) cyclotron frequency, Ω_ci_, and for short perpendicular wavelength, *k*_⟂_*V*_A_/Ω_ci_ ≫ 1, the fast MHD mode is decoupled. The perpendicular response is mainly due to the ions and the parallel response is mainly due to the electrons. The determinant of the dispersion relation can be written as

$$n_{\|}^{2}=S\left(1-\frac{n_{⊥}^{2}}{P}\right)$$ (7)

$$S=1+\frac{c^{2}}{V_{A}^{2}}G\left(\mu_{i},\omega /\Omega_{\text{ci}}\right)$$ (8)

$$P=1+\frac{\Gamma_{0}\left(\mu_{e}\right)}{k_{\|}^{2}\lambda_{D}^{2}}\left(1+\xi Z\left(\xi \right)\right)$$ (9)

where ξ = ω/*k*_||_*a*_e_, μ_i,e_ = *k*_⟂_^2^ρ_i,e_^2^, *a*_e_ = (2*T*_e_/*m*_e_)^½^, λ_D_ = (ε_0_*T*_e_/*ne*^2^)^½^, and ρ_i_ = (*m*_i_*T*_i_)^½^/*eB*.

$$G=\frac{1-\Gamma_{0}\left(\mu_{i}\right)}{\mu_{i}}+\frac{2\Gamma_{1}\left(\mu_{i}\right)}{\mu_{i}}\frac{\omega^{2}/\Omega_{\text{ci}}^{2}}{1-\omega^{2}/\Omega_{\text{ci}}^{2}}$$ (10)

where Γ*_n_*(*x*) = *e*^–*x*^*I_n_*(*x*) is related to the modified Bessel function and *Z*(ξ) is the plasma dispersion function. The dispersion relation to be solved is written in the form

$${\left(\frac{\omega }{k_{\|}c}\right)}^{2}=\frac{1}{1+\frac{c^{2}}{V_{A}^{2}}G}+\frac{k_{⊥}^{2}\lambda_{D}^{2}}{k_{\|}^{2}\lambda_{D}^{2}+\Gamma_{0}\left(\mu_{e}\right)\left[1+\xi Z\left(\xi \right)\right]}$$ (11)

The complex roots of this equation are found using Muller’s method. The real part of the complex root is the frequency and imaginary part is the growth rate. The electron beam is modeled as a drifting Maxwellian with a density that is 1% that of the plasma density. The results are weakly sensitive to the beam density. It is the plasma conditions, namely, temperature, density, and magnetic field, as well as the beam energy that principally control the dynamics.
