# Supplementary material for: Jupiter’s brightest auroras are powered by locally generated Alfvén waves
Source: Sci Adv. 2026 Sep 16;12(38):eaeg6021. doi: 10.1126/sciadv.aeg6021 (PMC13580586; doi:10.1126/sciadv.aeg6021)
Supplement: Supplementary file 1 [file sciadv.aeg6021_sm.pdf]

Science Advances
AAAS

Supplementary Materials for

Jupiter's brightest auroras are powered by locally generated Alfvén waves

Ali H. Sulaiman *et al.*

Corresponding author: Ali H. Sulaiman, asulai@umn.edu

Sci. Adv. **12**, eaeg6021 (2026)
DOI: 10.1126/sciadv.aeg6021

This PDF file includes:

Figs. S1 and S2

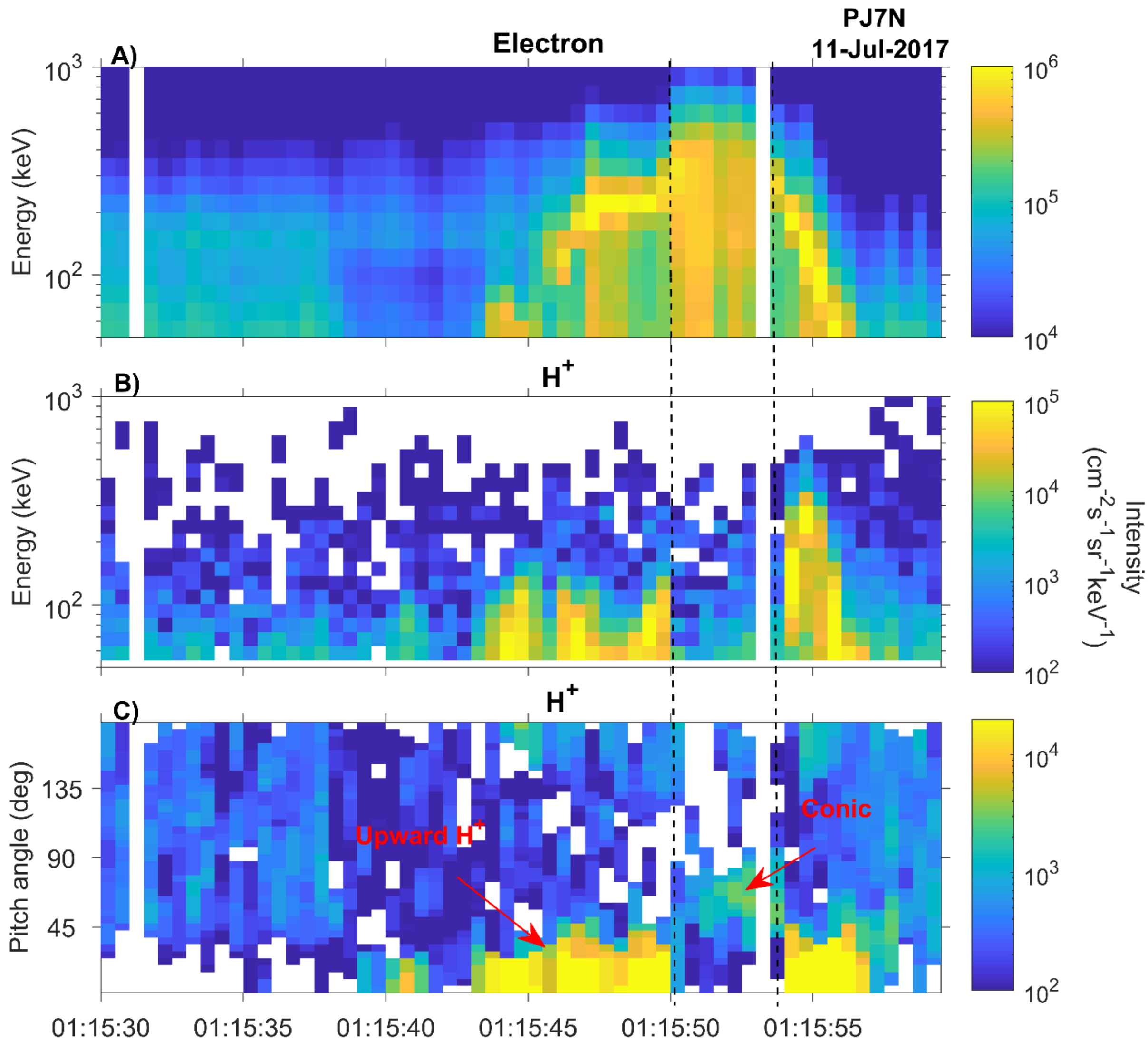

Fig. S1.

Energetic electron and H^+ spectra for PJ7N event. (A) Energy-time spectrogram of energetic electrons in the loss cone measured by JEDI during PJ7N. (B) Energy-time spectrogram of energetic H^+. (C) Pitch-angle time spectrogram of H^+. Broadband acceleration event is delimited by dashed lines.

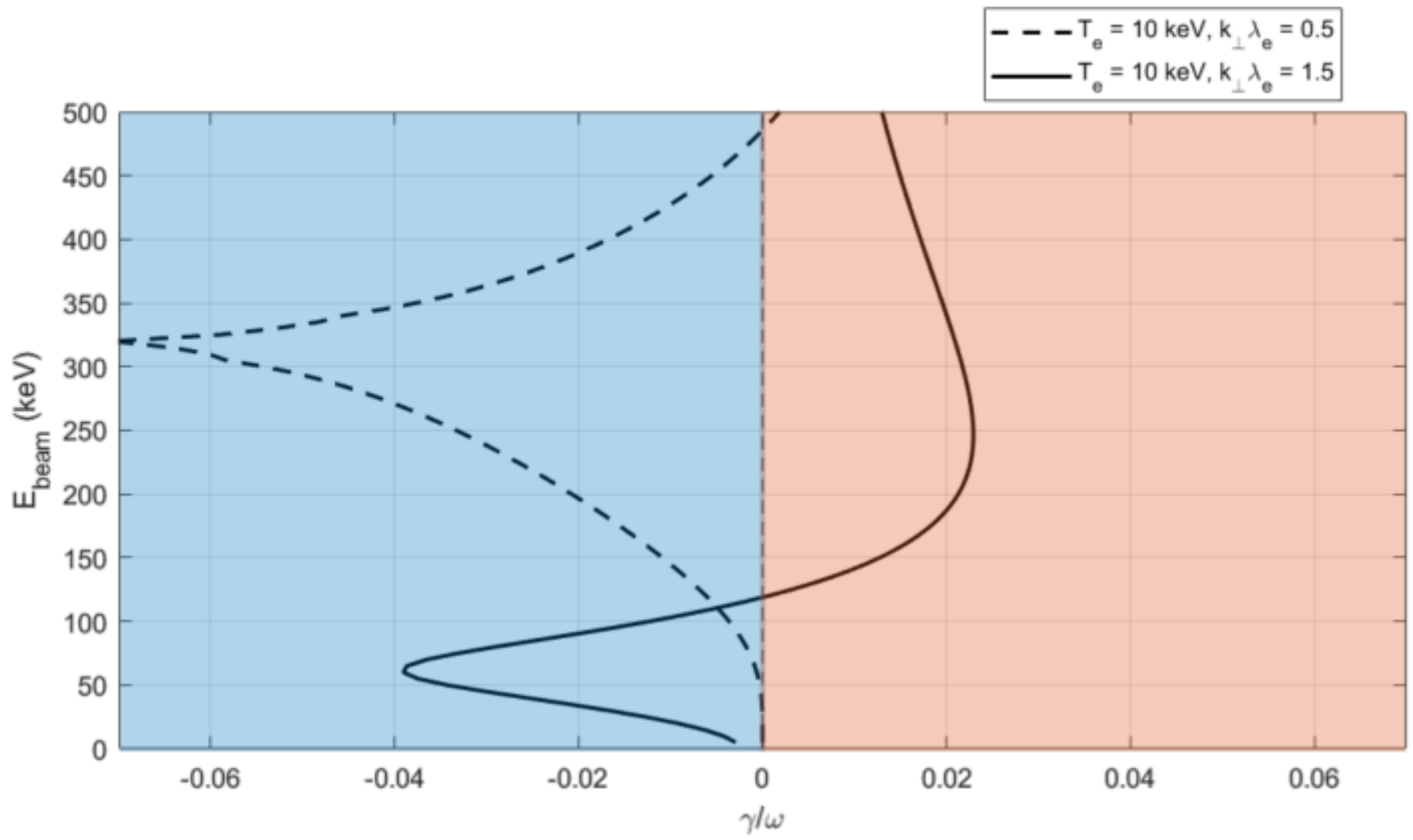

Fig. S2.

Normalized growth rates for PJ4S event. Beam energy versus normalized growth rates for T_e = 10 keV to represent PJ4S. The red region is that of instability (positive growth rate).
